# MrBayes tgMC^3^: A Tight GPU Implementation of MrBayes

**DOI:** 10.1371/journal.pone.0060667

**Published:** 2013-04-09

**Authors:** Cheng Ling, Tsuyoshi Hamada, Jianing Bai, Xianbin Li, Douglas Chesters, Weimin Zheng, Weifeng Shi

**Affiliations:** 1 Guangzhou Institute of Advanced Technology, Chinese Academy of Science, Guangzhou, China; 2 Department of Computer and Information Science, Nagasaki University, Nagasaki, Japan; 3 University of Chinese Academy of Sciences, Beijing, China; 4 Key Laboratory of Zoological Systematics and Evolution, Institute of Zoology, Chinese Academy of Sciences, Beijing, China; 5 School of Basic Medical Sciences, Taishan Medical College, Taian, Shandong, China; Institute of Infectious Disease and Molecular Medicine, South Africa

## Abstract

MrBayes is model-based phylogenetic inference tool using Bayesian statistics. However, model-based assessment of phylogenetic trees adds to the computational burden of tree-searching, and so poses significant computational challenges. Graphics Processing Units (GPUs) have been proposed as high performance, low cost acceleration platforms and several parallelized versions of the Metropolis Coupled Markov Chain Mote Carlo (MC^3^) algorithm in MrBayes have been presented that can run on GPUs. However, some bottlenecks decrease the efficiency of these implementations. To address these bottlenecks, we propose a tight GPU MC^3^ (tgMC^3^) algorithm. tgMC^3^ implements a different architecture from the one-to-one acceleration architecture employed in previously proposed methods. It merges multiply discrete GPU kernels according to the data dependency and hence decreases the number of kernels launched and the complexity of data transfer. We implemented tgMC^3^ and made performance comparisons with an earlier proposed algorithm, nMC^3^, and also with MrBayes MC^3^ under serial and multiply concurrent CPU processes. All of the methods were benchmarked on the same computing node from DEGIMA. Experiments indicate that the tgMC^3^ method outstrips nMC^3^ (v1.0) with speedup factors from 2.1 to 2.7×. In addition, tgMC^3^ outperforms the serial MrBayes MC^3^ by a factor of 6 to 30× when using a single GTX480 card, whereas a speedup factor of around 51× can be achieved by using two GTX 480 cards on relatively long sequences. Moreover, tgMC^3^ was compared with MrBayes accelerated by BEAGLE, and achieved speedup factors from 3.7 to 5.7×. The reported performance improvement of tgMC^3^ is significant and appears to scale well with increasing dataset sizes. In addition, the strategy proposed in tgMC^3^ could benefit the acceleration of other Bayesian-based phylogenetic analysis methods using GPUs.

## Introduction

In biology, the evolutionary relationships between groups of organisms or families of related genes and proteins can be inferred from the pattern of states at homologous characters, and represented as a tree structure. A number of algorithms have been developed to construct phylogenetic trees, such as Neighbour-Joining (NJ) [1] or Maximum Parsimony [2], whereas methods that implement a model of sequence evolution, such as Maximum Likelihood [3] and Bayesian inference [4], are more computationally demanding [5]. MrBayes [6] is a popular tool that implements the Metropolis Coupled Markov Chain Monte Carlo (MC^3^) sampling method for Bayesian inference of phylogeny. Since each chain in MC^3^ runs more or less independently, MrBayes is well suited to parallel implementation on multi-core systems as an approach to reduce computation time.

The programmable Graphics Processing Units (GPUs) have become very powerful many-core processors, driven by demand from various graphical, computational and engineering applications. Current GPUs can attain a peak float-point throughput of up to 3250 GFlop/s per chip, which is higher than that of the fastest CPU by a factor of over 10. Moreover, recent GPUs support Compute Unified Device Architecture (CUDA) [7], which allows users to develop a wide range of applications under a high-level, general-purpose programming model. Such a general-purpose GPU computing (GPGPU) model significantly improves development productivity as it follows the traditional software programming flow. CUDA is the programming environment for NVIDIA GPUs. CUDA C is an extension of C programming language and follows an SPMD execution model with a thread as the basic unit for parallel computations. Tens of thousands of threads can be launched concurrently, all executing the same program, but on different data packets. Threads in the same block can share data using fast on-chip memory (shared memory). Threads in different blocks can only share data using relatively slow on-board memory (global memory) on a GPU card. During execution, every 32 threads in a block (a warp) follow exactly the same instruction schedule.

To increase efficiency of GPU implementation of MrBayes MC^3^, we propose a new implementation architecture, tgMC^3^. A performance comparison between tgMC^3^, BEAGLE-based MrBayes MC^3^ [8], nMC^3^ (v1.0) [9], nMC^3^ (v2.1, the latest version so far), as well as the multi-core parallel MrBayes MC^3^, is performed.

## Approach

### Overview of MrBayes MC^3^

**Figure 1:**
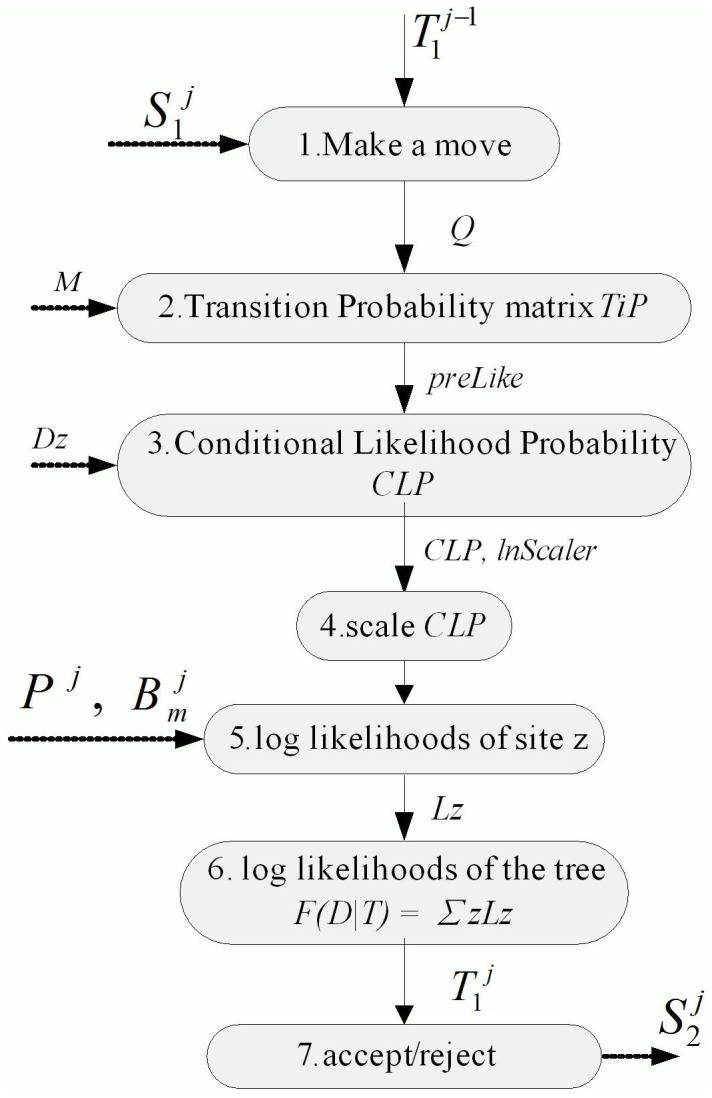
The flow diagram of MrBayes MC^3^. The gray box indicates the detailed operation of each procedure, and the arrow indicates the required parameters in a given procedure.

MrBayes MC^3^ is a computer package for the Bayesian inference of phylogeny, which has a command-line interface and performs phylogenetic analysis under various evolutionary models. For the purpose of a standardized evaluation against other implementations, we used MrBayes v3.1.2 (unless stated otherwise) under a commonly applied model which is set using the following command:
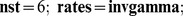



A detailed description of MrBayes MC^3^ settings can be found elsewhere [10], but briefly, this model allows gamma distributed variation in the rate of substitution over sites [11], with six substitution rate parameters, four base frequencies, and a proportion of invariable positions. Both the sampling frequency and the diagnosis frequency were set to 1000. The total number of generations for each dataset in our experiments can be found in section 4. For each dataset, two independent runs were performed, each with four Markov chains (three heated chains and one cold chain). Multiple chains can be used for fast mixing [12]. For individual run, each chain is operated one by one as depicted in Figure 1. We let *T_N_* denote the initial tree of chain *N*, 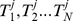 denote the new group of trees modified from 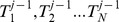 by proposed moves in iteration *j*, 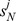
denote the seed value of chain *N* in iteration *j* and *Q* denote whether the move is aborted.

For each chain, we let *M* denote the mutation matrix, *D_z_* denote the DNA data at site *z*, *L_z_* denote the number of site *z*, *P^j^* denote the proportion of invariable sites and 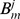 denote the base frequency of character state in DNA sequence in iteration *j*, where *m∈{A, C, G, T}.* The computation order of nodes in MrBayes MC^3^ uses the recursive algorithm [3]. In each procedure, the parameter *Q* needs to be verified in advance.

Since Markov chains in MrBayes MC^3^ are scheduled serially, and the run-time of each tree can be considered identical, the elapsed time can be regarded proportional to the number of chains. In addition, the number of unobserved tree nodes in an arbitrary tree topology and the computations of conditional likelihood probabilities of non-terminal node in each chain can be deemed as constant and performed independently.

### Overview of Parallel MrBayes MC^3^ on GPUs

To our knowledge, the first parallel version of MrBayes MC^3^ was gMC^3^ [13], [14]. Since the transfer of the transition probability (*tip)* matrix between CPU and GPU is very frequently, it meets a large transfer overhead. Based on the gMC^3^, an improved parallel version of MrBayes MC^3^ was proposed (nMC^3^ [9]). In the initial version of nMC^3^, the authors decreased the frequency of *tip* matrix uploading and made both the CPU and GPU perform computations in parallel. This results in improved overlap in CPU-GPU data communication. In addition, site likelihood computation was performed in parallel by GPUs, which gives a delay in GPU to CPU transfer and decreases the amount of data transferred. Further speedups were achieved as a result of these modifications. In subsequent versions of nMC^3^, the authors optimized the stream order and thread parallelization strategy for large datasets. However, these various nMC^3^ implementations all used traditional one-to-one acceleration architecture, thus giving complex kernel launches. For the purpose of avoiding redundant computations and data transfer, we propose a new improved architecture for applying MrBayes MC^3^ to GPUs. Furthermore, Ayres and colleagues implemented an open API library, BEAGLE, to speed up likelihood calculations [8]. The BEAGLE library is now supported in MrBayes (v3.2.1), and is also included in this article for the performance comparisons.

## Design and Implementation

### 1. The MrBayes nMC^3^ Method

The two most time-consuming aspects of MrBayes MC^3^ are the calculation of *tip*, and the calculation of *clp.* Since the implementation of the former on GPUs is identical in both tgMC^3^ and nMC^3^, we will not introduce this process here (for more details, see [9]).

**Figure 2:**
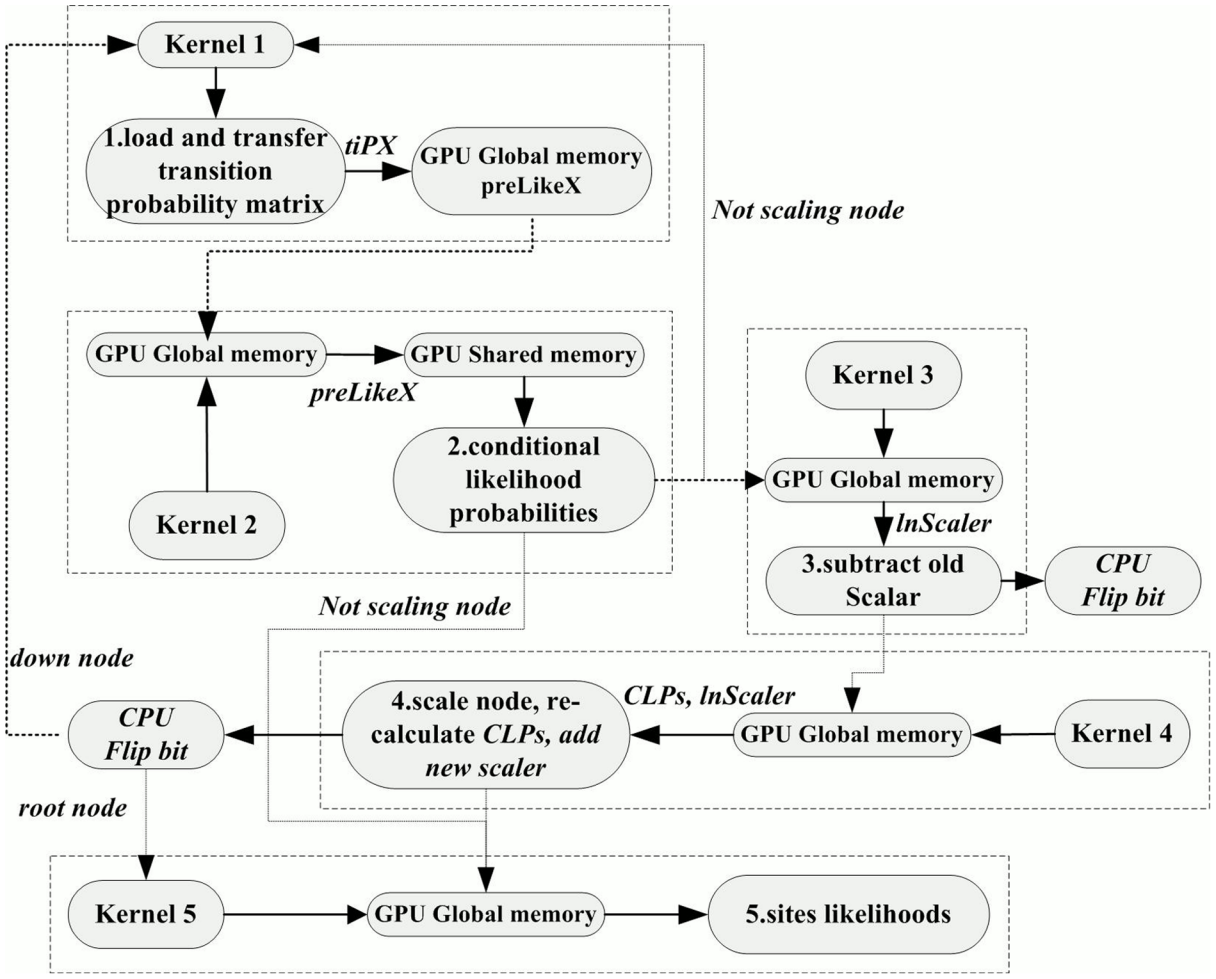
GPU implementation architecture in nMC^3^. Each module is implemented by a single kernel which is labeled in a rectangle by dashed line.

Whereas GPU computation of *clp* under nMC^3^ is as follows. We assume that the transition probability matrix for each non-terminal node has already been calculated and transferred to GPU memory. As in Figure 2, the implementation architecture of nMC^3^ can be grouped into five modules according to the associated procedures in MrBayes MC^3^, with each module implemented by a single kernel which is labeled in a rectangle by dashed line. We will first introduce the essential function of each kernel, and then give the potentially redundant procedures in nMC^3^ by discussing the data relevance among the kernels.

#### kernel1

Transform *tip* matrix of non-terminal nodes and pad the transformed matrix (*prelike* matrix) with new *tip* for ambiguity data in parallel by GPU threads. Not all non-terminal nodes need to perform the procedure, as some of them may not have terminal node.

#### kernel2

Load *tip* or *prelike* matrix from global memory to shared memory, in addition to terminal states or *clp* of its descendents, and distribute the computation of *clp* among threads. The computed *clp* values need to be saved in global memory iteratively until reaching the root node.

#### kernel3

If the parameter *scalarsSet* of the node is activated, the *lnscaler* value of each site belonging to the node should be subtracted by old scaler, with kernel 3 used to implement this function. In the mean time, the CPU process flips the *scalersSet* bit alongside the GPU.

#### kernel4

For a scaler node, MrBayes MC^3^ stipulates the maximum *clp* within each residue as the *scaler* of the residue, which is achieved simply by traversing all *clp*. The new scaler is then divided by all *clp* of that residue. The kernel employs multiple threads to traverse the corresponding *clp* and scale them in parallel, which requires reloading *clp* from global memory. Similarly, alongside the GPU, the CPU process flips the *scalersSet* bit.

#### kernel5

The kernel computes the *clp* of a root node, which requires the following new parameters: proportion of invariable sites, state frequencies and the weight of each site.

Kernels 3 and 4 may not occur in some non-terminal nodes. Therefore, if the node is non-scaling, only kernels 1, 2 and 5 will be implemented for a root node, and 1, 2 will be implemented for down nodes. Otherwise, the number of kernels for root and down node is 5 and 4, respectively. Besides, kernels 3 and 4 are two independent procedures. The bit switch operation of the former will not affect the computation of the latter, and the updated value only affects the computation of the new tree sample in the next generation. Therefore, kernels 3 and 4 can be merged into one module and the bit switch operations can be stacked and implemented together by CPU processes after these modules. Moreover, new parameters in kernel 5 can be calculated in advance and merged in kernel 2 for computing likelihoods of the root node.

### 2. An Improved GPU Implementation of MrBayes MC^3^

**Figure 3:**
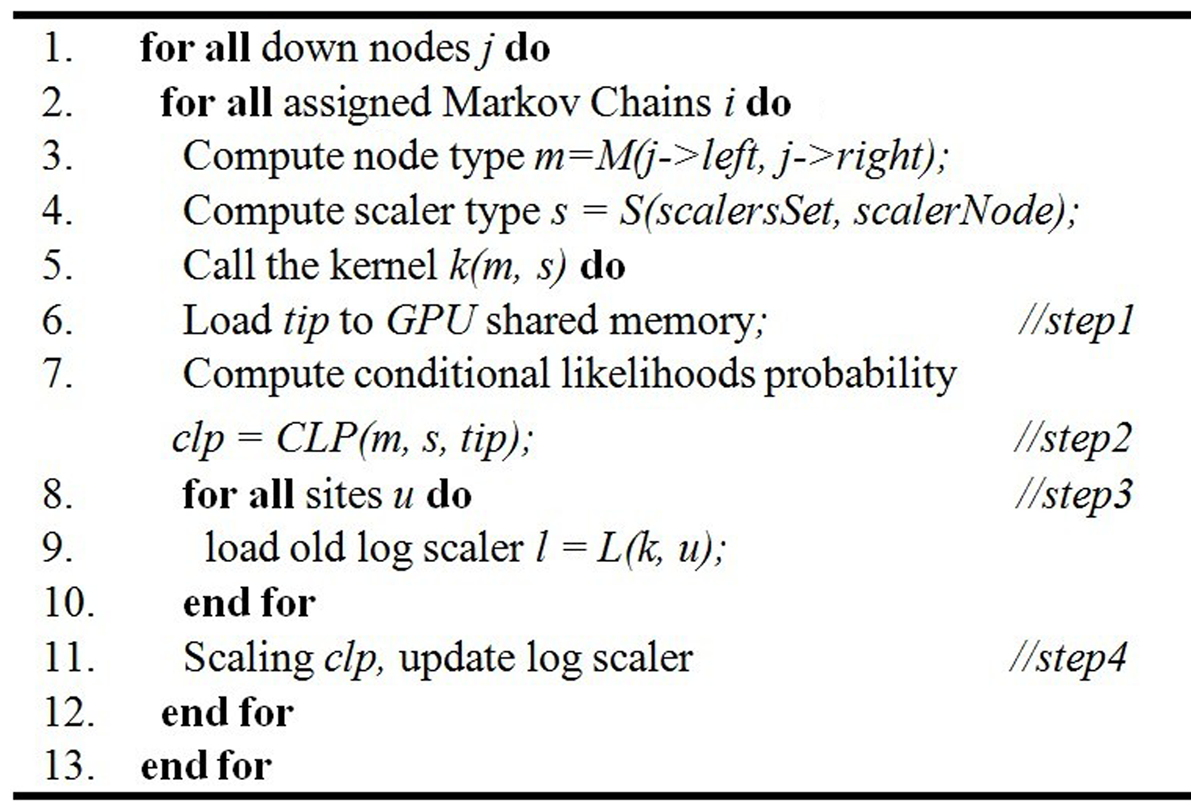
Pseudo code of computing the conditional likelihood probability of down nodes implemented in tgMC^3^.

**Figure 4:**
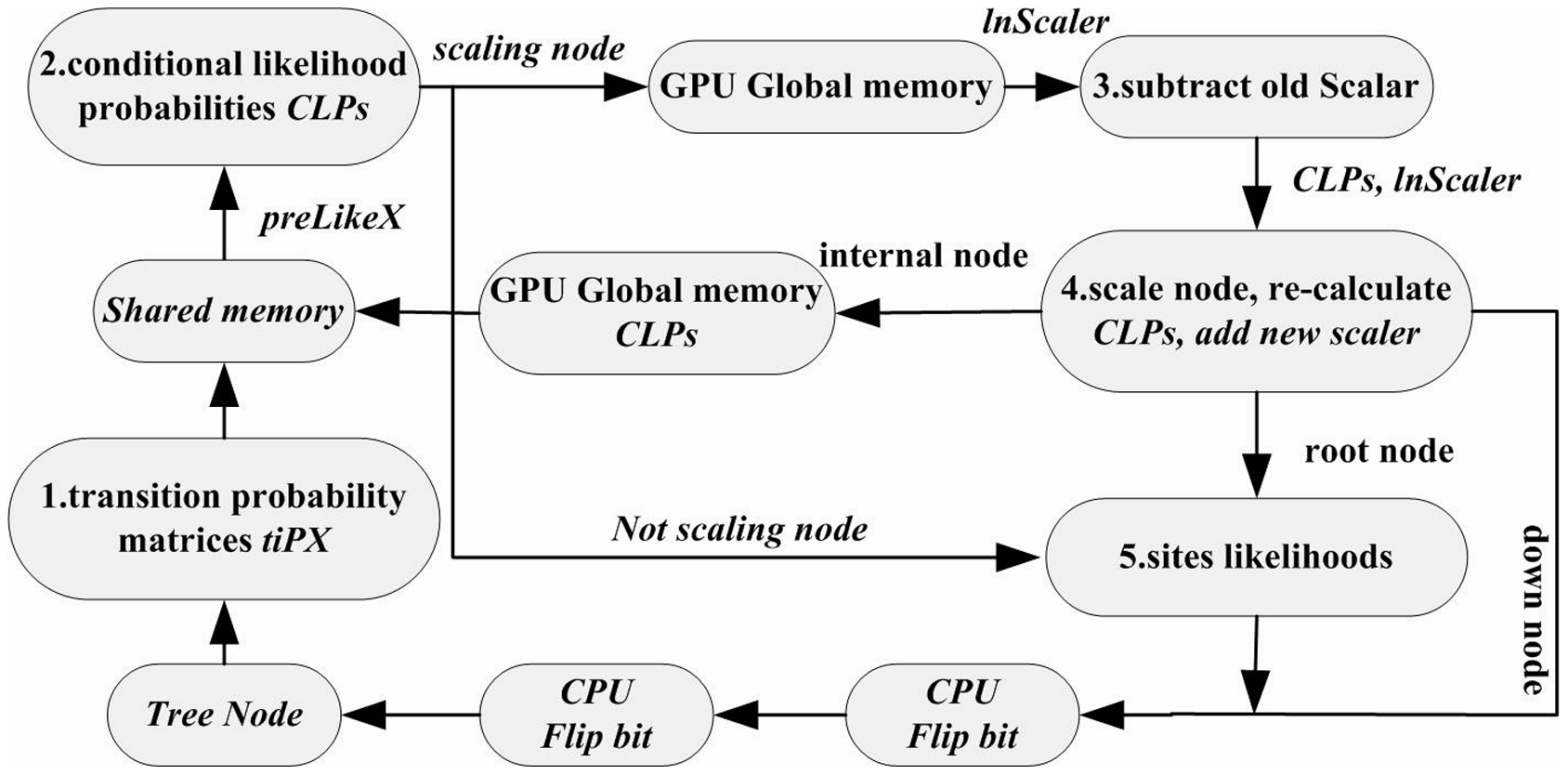
The proposed tight GPU (tgMC^3^) implementation architecture.

Since the computation of *clp* can be regarded as a pipeline of several sequential steps, we put forward a tight GPU implementation of MrBayes MC^3^, tgMC^3^. The pseudo-code description of the *clp* computation for an arbitrary down node can be found in Figure 3. The single tight kernel integrates all kernel functions in earlier applications on GPUs. In tgMC^3^, we do not use global kernels to perform each function, but use device function in the kernel to process each step. The concise and explicit architecture of tgMC^3^ is illustrated in Figure 4. The major advantages of tgMC^3^ over nMC^3^ are listed below:

- By analyzing data dependencies in MrBayes MC^3^, tgMC^3^ integrates multiple functions into a single tight GPU kernel, instead of using several discrete kernels, hence decreasing the complexity of kernel launching. Just one kernel is sufficient for likelihood computation of both down and root nodes. This is particularly useful for large datasets having a relatively small amount of unique sites.
- In step 1, for terminal nodes, nMC^3^ loads *tip* and the transition probability for ambiguity data initially into a *prelike* matrix, and then re-load the *prelike* matrix from global memory into shared memory for likelihoods computation. However, this is a redundant process on GPUs, which we have improved here. A direct multi-threaded data transfer of *tip* matrices is proposed in the tgMC^3^ method.
- For scaling nodes, the nMC^3^ method accelerates steps 3 and 4 by two individual GPU kernels. However, the scaled data and scaler are closely connected with conditional likelihoods from step 2. Therefore, combined with the original down node types, a scaler shortcut is calculated by scaler parameters for the purpose of integrating these steps without adding branches, and the final number of down node kernel type is expanded from 4 to 16. These modifications improve the scaling node procedures on GPUs.

#### 2.1 A direct read of transition probability matrix

In MrBayes MC^3^, in the *j^th^* generation of Markov chain *i*, on the newly proposed tree topology *T*, for non-terminal node *k*, site *l*, discrete rate *r* and nucleotide *m*, where *m∈{A, C, G, T},* before computing a conditional likelihood probability *clp_m_ = clP(T, k, l, r, m)* of the node, the corresponding transition probability matrices *tip* for terminal node descendents should be transformed. Since each non-terminal node has two child nodes which are either terminal or non-terminal, non-terminal node can be classified into 4 groups. MrBayes MC^3^ assesses the combinations of the node, and labels the node as one of *down_0*, *down_1*, *down_2* or *down_3*. The computation of *clp* is then distributed to the relevant pass.

In the nMC^3^ algorithm, the procedure described above is split into two sections by first accelerating the transformation of *tip* matrices to *prelike* matrices, and then accelerates the computation of *clp*. These accelerations are implemented by two separate kernels. Assuming that both the left and right children of a node are terminal, i.e. the node belongs to *down_3*, *tipL* and *tipR* matrices need to be transformed into *prelikeL* and *prelikeR* matrices. For this, the implementation requires two global memory reads and writes. Since *prelike* matrices are in use in the computation of *clp*, one additional global memory read is needed.

In the tgMC^3^ algorithm, to compute *clp = CLP(T, k, l, r, m)* over all *l, r* and *m* at non-terminal node *k* on the newly proposed tree topology *T*, nodes will be distributed into the relevant *down_x*, *x∈ {0, 1, 2, 3}* after assessment. The implementation is performed by transforming the *tipL* and *tipR* matrices to *prelikeL* and *prelikeR* matrices in shared memory directly for the computation of *clp*, instead of loading from global memory repeatedly. Hence, the whole procedure is performed within the same kernel on GPUs.

#### 2.2 One tight kernel versus multiple discrete kernels

Some non-terminal nodes are specified to be scaled after the *clp* of these nodes are computed. In MrBayes MC^3^, scaling is performed either by subtracting the value of the old scaler or adding the new scaler to the *lnScaler* variable, or both steps are performed successively. These steps are followed by two bit switch operations. These operations actually block the scaling being performed in the same kernel on GPUs in the nMC^3^ algorithm. As explained in section 3.1, these steps are independent and the bit switch operation of step 1 will not affect the input variables of step 2. Hence they can be grouped and performed after the two steps. Experimental results confirm that the accuracy of the results is not changed by porting these codes (Table S1). This principle is adopted for our tgMC^3^ algorithm. Since the two steps may not occur on the same node, simply including these steps in the kernel will result in redundant branch judgments for GPU threads. Therefore, we develop a shortcut list helping the CPU decide which tight GPU kernel to be launched (Table 1), where the values of *S* and *N* represent whether *scalersSet* or *scalerNode* is activated. *T* denotes the shortcut for each type of down node, i.e. a secondary classification of down nodes. With these improvements, a union implementation scheme is established so that the whole implementation architecture employs just one tight GPU kernel. This not only decreases the CPU-GPU communication overhead, but also affords new implementation architecture to avoid redundant data transfers. In particular, the tight GPU kernel is extremely useful for *clp*, as shared memory and registers can be fully utilized, instead of repeatedly loading the data from global memory.

**Table 1:** Shortcut list of the tight GPU kernel.

S	N	T	down_0	down_1	down_2	down_3
0	0	0	down_0_0	down_1_0	down_2_0	down_3_0
0	1	1	down_0_1	down_1_1	down_2_1	down_3_1
1	0	2	down_0_2	down_1_2	down_2_2	down_3_2
1	1	3	down_0_3	down_1_3	down_2_3	down_3_3

Table 2 presents the data transfer complexity of the major parameters in the GPU-based implementation of MrBayes MC^3^, where the read and write operations on global memory are separately counted. Since the types of node and the scaler state are indeterminate, it is not feasible to calculate a completely accurate value. Hence, the statistics of data transfers are established in the case that each non-terminal node is composed of a terminal node and a non-terminal node descendent, and that all nodes are scaling-node. If the size per transfer is normalized to 1, transfer complexity can be decreased from *O(4(N-2)+5) to O(2(N-2)+2)* for *clp*, from *O(3(N-2)+3)* to *O(N-1)* for *tip* and from *O(4(N-2)+4)* to *O(2(N-2)+2)* for *lnscaler*. However, we will not claim that the improved method can achieve such improvements in practice, and the aim of such a comparison is primarily to highlight the differences between tgMC^3^ and nMC^3^.

**Table 2:** The complexity of data transfers in nMC^3^ and tgMC^3^ *.

	nMC^3^		tgMC^3^	
	down node	root	down node	root
clp→g	2(N-2)L*d(clp)*	2L*d(clp)*	(N-2)L*d(clp)*	L*d(clp)*
clp←g	2(N-2)L*d(clp)*	3L*d(clp)*	(N-2)L*d(clp)*	L*d(clp)*
tip→g	(N-2)*d(tip)*	*d(tip)*	*0*	*0*
tip←g	2(N-2)*d(tip)*	2*d(tip)*	(N-2)*d(tip)*	*d(tip)*
ls→g	2(N-2)L*d(f)*	2L*d(f)*	(N-2)L*d(f)*	L*d(f)*
ls←g	2(N-2)L*d(f)*	2L*d(f)*	(N-2)L*d(f)*	L*d(f)*

* N, number of taxa;

N-2, number of down node;

L, number of sites, i.e. length of compressed DNA sequences;

g, GPU global memory, →g denotes write operation on g and ←g denotes read operation on g;

ls, lnScaler;

*d(x)*, memory space used to store x. Particularly, *d(f)* is memory space used to store a float variable, so d(f) is 4 bytes. A residue in *d(clp)* is 16*d(f)*, and *d(tip)* is 64d(f) bytes.

#### 2.3 Intra-task parallelization versus Inter-task parallelization

The proposed algorithm is implemented by two task parallelization strategies. The difference between these strategies is whether the computation of an individual nucleotide residue is performed by a single thread or by multiple threads. An intra-task parallelization strategy is normally used to process relatively short nucleotide sequences in order to utilize GPU resources to the greatest extent, whereas shared memory is commonly exploited for faster accession of data. The inter-task parallelization method is normally used to process relatively long nucleotide sequence, as the overlap of memory access is higher, which also reduces costs on dispatching threads. In the specified evolutionary model, 16 elements for each nucleotide need to be calculated in total, which is separated into 4 sets, each with four values corresponding to the *A, C, G, T* nucleotide states. In nMC^3^ (v1.0), this is performed by a single parallelization strategy, the so-called Intra-task parallelization method, where the computation of each nucleotide residue is completed by 16 threads. In nMC^3^ (v2.1), the situation appears quite complicated, as the module for computing *clp* (kernel 2) is performed by mixed parallelization strategies (Figure 2), where the inter-task parallelization strategy is directed against long sequences and the intra-task parallelization strategy is directed against short sequences. The module for scaling nodes (kernel 4) is performed simply using the inter-task parallelization strategy. The parallelization strategy used in version 1.0 is suitable for short sequences, since if there are only a few nucleotide residues, increased thread allocation makes efficient use of GPU resources, although there is cost in terms of coordination among threads. The fact that version 1.0 adopts a reduction method to find the maximum *clp* value of each nucleotide residue in module 4 results in 50% of threads working in an idle state during each iteration. Nonetheless, Intra-task parallelization is still more preferred than Inter-task parallelization on the implementation of relatively short sequences. For version 2.1, module 4 can be performed by two parallelization strategies, which we improved here. Therefore, tgMC^3^ contains two parallelization strategies, and a threshold value regarding to the choice of parallelization strategy is empirically defined before running.

## Experiments

### 1. Experimental Environments

All experiments were benchmarked on the same computing node of DEGIMA (Destination for Gpu intensive MAchine) [15], built by one of the authors of this article (TH) for high performance computing research in Nagasaki University. The process technology of the CPU used in [9] is less current than the GTX 480 card, and hence we used a relatively advanced processor based on 32 nm process technology for equivalent comparisons. The details of experimental environments are listed in Table 3. The gcc version 4.4.4 with the –O_3_ and *–*Wall flag was used for compiling MrBayes MC^3^, as well as the CPU-side code of tgMC^3^, nMC^3^ and BEAGLE-based MrBayes. The GPU-side code of tgMC^3^, nMC^3^ and BEAGLE-based MrBayes MC^3^ were compiled using CUDA Toolkit, version 4.2. *CUDA_INSTALL_PATH* and *SDK_INSTALL_PATH* should be imported to environment before making the codes, more details can be found in the user manual of tgMC^3^ (File S1).

**Table 3:** The experimental environments of host and device in the proposed method.

Host	GPU device
*Operating system*: Fedora Release 12*CPU*: Intel i7-3820 (4 cores, 3.6 GHz)*Memory*: 16 GB	*Graphic Driver:* NVIDIA Driver version 4.2*GPU*: NVIDIA GeForce GTX 480 (15×32 cores, 1.4 GHz)*GPU memory:* 1.5 GB

### 2. Datasets

The nucleotide datasets used in experiments can be placed into three categories according to their unique sites: short (datasets 1 and 2), medium (dataset 3) and long sequence length (datasets 4 and 5). We briefly list them below, and the details of these datasets can be found in [16]–[18].

Dataset 1: A group of Trichophora 18S rDNA including 26 taxa.

Dataset 2: A group of Euhemiptera 18S rDNA including 33 taxa.

Dataset 3: A group of metazoan 18S rDNA including 111 taxa.

Dataset 4: A group of eukaryotic 18S rDNA including 234 taxa.

Dataset 5: A group of 23 - 28S rDNA from Bacteria, Archaea, and Eukaryota including 288 taxa.

### 3. Results

#### 3.1 Run-time

The run-time gives elapsed time for the whole analysis. Table 4 presents the run-time required to analyze the datasets on the platforms described in Section 4.2 with 1) serial MrBayes MC^3^ using one CPU process, 2) MrBayes MC^3^ using two concurrent CPU processes, 3) BEAGLE-based MrBayes MC^3^ using one GTX 480 card, 4) nMC^3^ (v1.0 and 2.1) using one GTX 480 card, 5) tgMC^3^ using either one GPU or two GPUs. From Table 4 we can see that tgMC^3^ takes the least time in our test.

**Table 4:** Dataset information.

Dataset	No.of taxa	Alignmentlength (nt)	No.of generations	Run-time(s)						
				MrBayes1 core	MrBayes2 cores	BEAGLEwith1 GPU	nMC^3^(1.0) with1 GPU	nMC^3^ (2.1) with1 GPU	tgMC^3^with1 GPU	tgMC^3^with2 GPUs
1	26	1546	1,000,000	2485	1285	2338	1117	724	412	244
2	37	2238	1,000,000	8729	4529	3502	1653	1035	749	420
3	111	1506	500,000	10155	5178	3265	1870	1194	765	436
4	234	1790	100,000	7979	4050	1490	858	545	402	228
5	288	3386	100,000	17984	9101	2662	1492	734	610	352

#### 3.2 Speedup

We made the following definitions:

1. The *b-m speedup* is the number of times faster that BEAGLE-based MrBayes MC^3^ using one CPU process and one GPU device performs when compared with MrBayes MC^3^ implemented by either one CPU process or two concurrent CPU processes.
2. The *n-m speedup* is the number of times faster that the nMC^3^ (v2.1) algorithm using one CPU process and one GPU device performs when compared with MrBayes MC^3^ implemented by either one CPU process or two concurrent CPU processes.
3. The *t-m speedup* is the number of times faster that the tgMC^3^ algorithm using one CPU process and one GPU device performs when compared with MrBayes MC^3^ implemented by either one CPU process or two concurrent CPU processes.

Table 5 presents the speedups, computed from the data in Table 4. The tgMC^3^ method outperforms the serial MrBayes MC^3^ by a factor between 6 to 30 times when using a single GTX480 card, whereas a speedup factor around 51 times can be achieved by using two GTX 480 cards on relatively long sequences (Table 4). The number of times faster between each GPU-based MrBayes MC^3^ can also be computed from Table 4. Experiments indicate that the tgMC^3^ method outstrips nMC^3^ (v1.0) with speedup factors from 2.1 to 2.7 times and nMC^3^ (v2.1) from 1.2 to 1.7 times. Moreover, tgMC^3^ outperforms the BEAGLE-lib based method from 3.7 to 5.7 times. For all the five test datasets, tgMC^3^ always achieves the greatest speedup factor when using one core or two cores (Table 5).

**Table 5:** Speedup comparisons to MrBayes MC^3^.

Dataset	b-m speedup		n-m speedup		t-m speedup	
	1-core	2-cores	1-core	2-cores	1-core	2-cores
1	1.06	0.55	3.43	1.77	6.03	3.12
2	2.49	1.29	8.43	4.44	11.65	6.05
3	3.11	1.59	8.51	4.34	13.43	6.77
4	5.36	2.72	14.64	7.43	19.85	10.07
5	6.76	3.42	24.5	12.4	29.5	14.92

#### 3.3 Scalability

**Figure 5:**
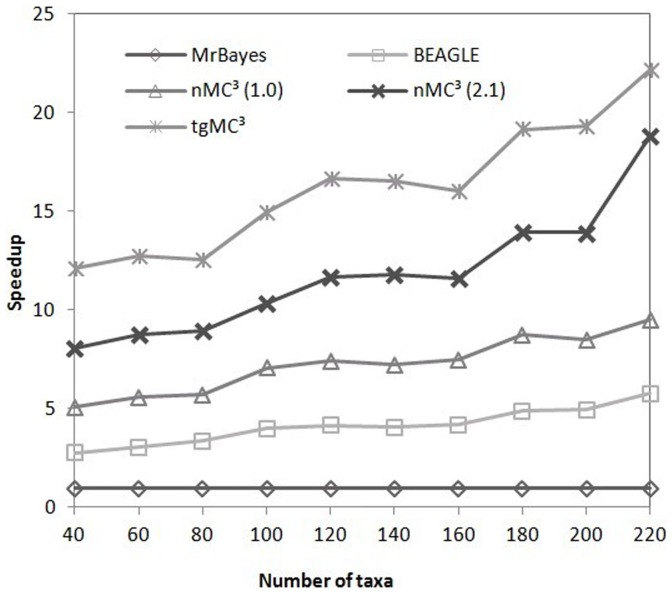
Speedup comparisons between BEAGLE, nMC^3^(v1.0), nMC^3^(v2.1) and tgMC^3^ on the first *N* taxa in dataset 4, where *N∈* {40, 60, …, 220}.

**Figure 6:**
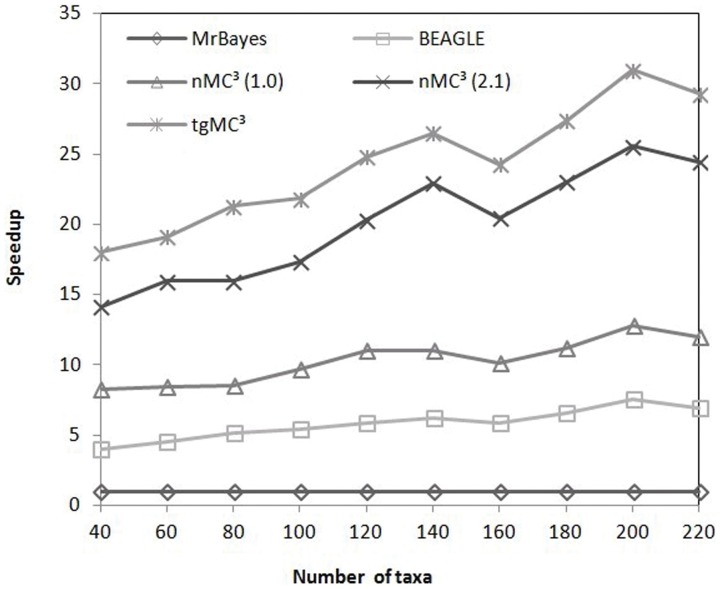
Speedup comparisons between, BEAGLE, nMC^3^(v1.0), nMC^3^(v2.1) and tgMC^3^ on the first *N* taxa in dataset 5, where *N∈* {40, 60, …, 220}.

**Figure 7:**
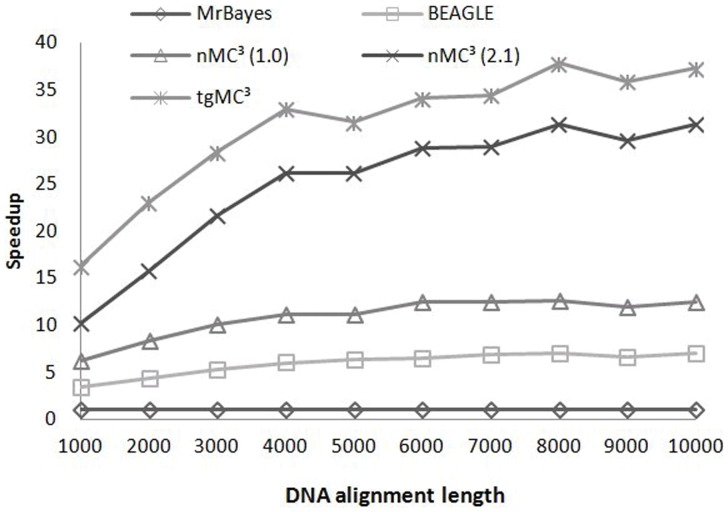
Speedup comparisons between BEAGLE, nMC^3^(v1.0), nMC^3^(v2.1) and tgMC^3^ on a group of 60 taxa, consisting *L* unique sites, where *L∈* {1000, 2000, …, 10000}.

The sequence length discussed in this section is the number of unique sites per dataset, as opposed to the complete length of sequences, since only unique sites affect the computation complexity in MrBayes MC^3^. We normalized the speedup comparisons of the GPU-based implementation of MrBayes MC^3^ to serial MrBayes MC^3^. Figures 5 and 6 illustrate the speedup comparisons between these methods when using the first *N* taxa of datasets 4 and 5. These datasets are composed of relatively long sequences, with 1790 nt and 3386 nt. From these figures, it is apparent that the speedup factors of tgMC3 and nMC3 (v2.1) show better scalability than the remaining methods. In particular, tgMC^3^ runs at least 1.2 times faster than nMC3 (v2.1). Figure 7 illustrates the speedup of a group of simulated datasets composed of 60 taxa, which were generated with Seq-Gen version 1.3.2 [19], consisting *L* unique sites. In each case, we run serial MrBayes MC^3^, BEAGLE, nMC^3^ (v1.0), nMC^3^ (v2.1) and tgMC^3^ for 10000 generations. The speedup of each GPU-version of MrBayes MC^3^ is normalized by the run-time of serial MrBayes MC^3^. Likewise, tgMC^3^ outperforms other methods and shows the best scalability as sequence length is increased (Figure 7).

## Discussion

While there have been a number of versions of MrBayes MC^3^ accelerated with GPUs, several bottlenecks still exist. Firstly, data transfer can impose a substantial overhead in GPU-CPU heterogeneous computing for MrBayes MC^3^ if calculation times are not well synchronized. Secondly, the adoption of several CUDA kernels to accelerate each sub-function in MrBayes MC^3^ can be inefficient. The use of multiple CUDA kernels may reduce speedup as there are redundant global memory accesses between kernels. Thirdly, the size of the input data influences the degree of acceleration, particularly with insufficient computational load reducing the efficiency of GPU hardware usage. Previous methods fail to take these into account.

To address these bottlenecks and further accelerate MrBayes MC^3^ on GPUs, we propose a tight GPU MC^3^ (tgMC^3^) algorithm with three key features; i) the algorithm applies a tight GPU kernel to avoid data transfer overhead between CPU and GPU; ii) it encapsulates conditional likelihood probability estimation in a single CUDA kernel, also reducing the run-time consumed on GPU device memory access; iii) it employs two different task parallelization strategies to make full use of GPU hardware resources. Thus, tgMC^3^ can outperform the serial MrBayes MC^3^ by considerable speedup factors on empirical and simulated datasets.

Figures 5 and 6 illustrate that the outperformance of tgMC^3^ compared to other acceleration methods with datasets of various size, although the scalability is similar to nMC^3^ (v2.1) for long sequences. tgMC^3^ and nMC^3^ (v2.1) also show similar scalability with the increase of alignment length (Figure 7). The reason is associated with the task parallelization strategies. In tgMC^3^, two different task parallelization strategies are used to accelerate the computation of *clp*. Intra-task parallelization is used for short and medium alignments, with the aim to fully utilize GPU resources by allocating as many threads as possible, whereas inter-task parallelization is performed for long sequences to avoid redundant computation. An alignment length threshold is defined to select amongst these two strategies. However, in nMC3 (v2.1), only inter-task parallelization is adopted. As datasets 4 (results shown in Figure 5) and 5 (results shown in Figure 6) are composed of long sequences, both tgMC3 and nMC3 (v2.1) apply the same strategy, inter-task parallelization. This accounts for similar performance with increasing alignment length.

As can be seen from Table 4, the performance of the BEAGLE-based (1 GPU) implementation and MrBayes MC^3^ (1 CPU core) is very similar when using dataset 1. Performance differs for all remaining datasets, with BEAGLE running in a much shorter time. While a GPU has many cores, the frequency of each core is relatively low. In cases where there is a small amount of data for computation, there is no advantage for parallel computing compared with serial computing. In addition, CPU-GPU data transfer could be a big overhead. If there is not enough data within each CUDA kernel or the data transfer frequency is continual, GPU-based heterogeneous computing is not a good option. BEAGLE-based implementation only parallelizes the computation of conditional likelihood probability, while the chains are still scheduled serially. Therefore, it cannot achieve much speedup on datasets with short alignments as there is insufficient computation load. When the alignment length increases, higher speedup can be achieved by BEAGLE. This also can be seen from the performance of other GPU-based implementations.

The experimental results show that the proposed tgMC^3^ algorithm achieves increasing speedup as the number of unique sites increases, compared with serial GPU-based MrBayes MC^3^. In addition, tgMC^3^ outperforms other GPU-based MrBayes MC^3^, by a speedup factor of 3.7–5.7 times to the BEAGLE-based method, and 2.1–2.7 times to the nMC^3^ algorithm (v1.0). Moreover, the tgMC^3^ algorithm achieves a speedup factor of up to 1.7 times compared with nMC^3^ (v2.1). A minimum speedup factor of 1.2 times can be achieved when computing sufficiently long sequences.

### Conclusions

We presented an accelerated implementation of MrBayes MC^3^ by employing an encapsulated GPU-based implementation architecture, tgMC^3^. Besides the acceleration strategies implemented in nMC^3^, the proposed tgMC^3^ further reduces the run-time of MrBayes MC^3^ by decreasing data transfer overhead, and accelerating the computation of conditional likelihood probabilities of sampling trees by two different task parallelization strategies. A number of empirical and simulated datasets are used to assess the speedup and scalability of the algorithm. The experiments indicate that one GTX 480 card can improve the performance of serial MrBayes MC^3^ running on a start-of-the-art general purpose processor by a factor of 6 for datasets with short sequences, and up to roughly 30 for datasets with long sequences. Moreover, tgMC^3^ outperforms the nMC^3^ algorithm (v1.0) from 2.1 to 2.7 times and the latest version (v2.1) from 1.2 to 1.7 times depending on the alignment length. In addition, tgMC^3^ shows similar scalability to the nMC^3^ algorithm (v2.1), with increasing data size and alignment length. To conclude, tgMC^3^ achieves better acceleration than previously proposed GPU-based optimization strategies of MrBayes MC^3^. In particular, our method could benefit the acceleration of the latest version of MrBayes (v3.2.1) and other Bayesian-based methods for phylogenetic analysis.

## Supporting Information

File S1 User manual.
(DOCX)

Table S1 Split frequency comparisons after resolving scaler code.
(XLSX)
