# Supplementary material for: MrBayes tgMC3: A Tight GPU Implementation of MrBayes
Source: PLoS One. 2013 Apr 9;8(4):e60667. doi: 10.1371/journal.pone.0060667 (PMC3621901; doi:10.1371/journal.pone.0060667)
Supplement: File S1 — User manual. (DOCX) [file pone.0060667.s001.docx]

MrBayes tgMC^3^ (version 1.0) User Manual

*Cheng Ling, Tsuyoshi Hamada, Jianing Bai*

*Xianbin Li, Douglas Chesters, Weimin Zheng, Weifeng Shi*

*Oct 21, 2012*

1. Introduction

MrBayes tgMC^3^ (version 1.0), a tight GPU implementation of MrBayes MC^3^, is a modified version of [MrBayes (version 3.1.2)](http://mrbayes.sourceforge.net/download.php) that enables accelerating Metropolis Coupled Markov Chain Monte Carlo (MC^3^) sampling on CUDA-compatible Graphics Processing Units (GPUs). MrBayes tgMC^3^ has not been designed to perform all features and settings that MrBayes has, it specifies a GTR + I and GTR + I + Γ model (a General Time Reversible model with a proportion of invariable sites and a gamma shaped distribution of rates across sites) for nucleotide sequences, which has the settings as

*nucmodel = 4by4;*

*nst = 6;*

*rates = gamma or invgamma;*

*datatype = dna;*

The implementation of MrBayes tgMC^3^ needs at least one CUDA-compatible GPU card. In addition, two software packages are required to been installed before the running, which are CUDA toolkit and GPU computing SDK. The release version can be downloaded from [here](https://developer.nvidia.com/cuda-toolkit-42-archive) (CUDA 4.2 for Linux).

1. Compile and implement MrBayes tgMC^3^
   1. Prerequisites

MrBayes tgMC^3^ supports concurrent execution of multiple CPU processes by using Message Passing Interface MPICH2 (version 1.1). Before using multiple CPU processes, the value of MPI variable in the make file should be changed to **YES**. Make sure import CUDA_INSTALL_PATH and SDK_INSTALL_PATH to environment variable list before issuing **make** command, otherwise, the OS may not find the correct compiler, CUDA-related libraries and head files. Type command **set** will display environment variable list in terminal.

By default, we assume that MrBayes tgMC^3^ is implemented on a machine with one core and one GPU, and the maximum number of GPU can be set in cuda.cu file. The number of CPU process should be equal to the number of GPU in use.

- 1. Installation of MrBayes tgMC^3^

Provided that the prerequisites have been met, MrBayes tgMC^3^ is ready to be installed.

Step1: Download the source code of MrBayes tgMC^3^. MrBayes tgMC^3^ for Linux is available from <http://code.google.com/p/a-tight-gpu-implementation-of-mrbayes/>, the name of the package is:

*tg_mrbayes_v1.0.zip*

Step2: Unzip the package and compile it as:

*unzip tg_mrbayes_ v1.0.zip*

*cd tg_mrbayes _v1.0*

*make clean*

*make*

- 1. Implement MrBayes tgMC^3^

Let DATA denote the name of the input data file, the immediate command

*./mb DATA*

results in running MrBayes tgMC^3^ by only one CPU process. For example,

*./mb dataset/dataset1(26x1546).nex*

For using multiple CPU processes, the command below will be initiated

*mpiexec –n <p> ./mb DATA*

Where <p> denotes the number of concurrent CPU processes (which should be no more than the number of CPU cores), for example,

*mpiexec –n 2 ./mb dataset/dataset1(26x1546).nex*

The file dataset1(26x1546).nex is one of the datasets used to benchmark MrBayes tgMC^3^ , which has been included in the downloadable MrBayes tgMC^3^ package.

We note that in the nMC^3^ algorithm, the authors did not track the AbortMove parameters for all chains, which might lead to the failure of the chains to converge after a long period of run. Hence, we modified the code. If you want to try the previous one, just comment the code #undef USE_NMC3 in the mcmc.c file, or comment the code #define USE_NMC3 if tracking is preferred.

MrBayes tgMC^3^ has been tested on GTX480 and GTX580. However, it may have problems when executed on other GPU platforms. If there are any bugs for MrBayes tgMC^3^, please do not hesitate to contact us. <c.ling@giat.ac.cn>, <wf.shi@giat.ac.cn>
